# Associations between perceived and actual physical literacy level in Chinese primary school children

**DOI:** 10.1186/s12889-020-8318-4

**Published:** 2020-02-10

**Authors:** Ming Hui Li, Raymond Kim Wai Sum, Cindy Hui Ping Sit, Stephen Heung Sang Wong, Amy Sau Ching Ha

**Affiliations:** The Chinese University of Hong Kong, Shatin, Hong Kong SAR China

**Keywords:** Perceived physical literacy, Physical literacy and physical activity, Associations, Primary school children

## Abstract

**Background:**

The concept of physical literacy (PL) is gaining popularity within public health and physical education circles. However, little is known about the relationship between perceived and actual PL levels among school-aged children. The aim of this study is to explore the associations between perceived and actual levels of PL of primary school students in China.

**Methods:**

A total of 327 children (153 boys and 174 girls) with a mean (SD) age of 10.0 (±1.0) years were included for analysis. PL perceptions were measured using the Perceived Physical Literacy Instrument. Children’s actual level of PL was objectively assessed by the Chinese version of the Canadian Assessment of Physical Literacy, 2nd edition, which consists of four domains: Daily Behavior, Physical Competence, Motivation and Confidence, and Knowledge and Understanding. Pearson’s correlation coefficients were calculated to examine the relationship between students’ perceived and actual PL levels, whereas Multivariate Analysis of Variance (MANOVA) was calculated to investigate the gender, relative age differences, and interaction effect (2 × 4) on perceived and actual PL levels respectively.

**Results:**

Significant correlations were observed between the perceptions and actual PL scores in both boys (*r* = .46, *p* < .01) and girls (*r* = .41, *p* < .01). Low to moderate significances were shown between each domain of perceived PL and actual PL (from .16 to .49). Further MANOVA results revealed that there were significant gender differences in the Daily Behavior domain of actual PL (*F* (1, 319) = 30.15, *p* < .001, Wilks’ Λ = 540.88, η^2^_partial_ = .09). Overall, boys had both higher actual PL scores (58.9) and PL perceptions (37.3) than girls. Neither relative age effect nor interaction effect (2 × 4) was observed for the current participants in all the variables.

**Conclusions:**

This study is the first to examine the associations between the perceived and actual levels of PL in Chinese children. Additional studies should explore the importance of children’s perceptions of PL when assessing the actual level of PL in physical education and health settings. Girls should be more encouraged by PE teachers to participate actively in physical activities in the school environment.

## Background

Physical literacy (PL) is considered as an important prerequisite for lifelong physical activity participation [1] and assessing standards for quality physical education [2]. The concept of PL is defined as the “motivation, confidence, physical competence, knowledge and understanding to value and take responsibility for engagement in physical activities for life” (p.12) [3]. As a multifaceted construct, PL has been explored within different epistemologies. This multidimensional concept emphasizes an individual can lead a fulfilling life encompassing “the motivation, confidence, physical competence, knowledge and understanding to maintain physical activity” through enriching embodied experiences [4]. PL nurtures the disposition of an individual to establish a purposeful physical activity for pursuing an active lifestyle [5]. A consensus on the importance of PL in promoting physical activity and active living is attained in several countries, such as Canada, Australia and the UK, to pioneer large-scale programs innovatively in public health settings [6]. Since PL has been gaining popularity in the areas of sports and health in response to the global decline of children’s participation in physical activity, many countries have incorporated the concept of PL into their educational systems to create a renewed focus on physical education in schools. For example, SHAPE America implementing national standards for health and PE replaced the term “physically educated” with “physically literate” in the K-12 Physical Education National Standards to emphasize the importance of maintaining lifelong physical activity for children [7]. Meanwhile, PL has worked as a guiding ideology for quality PE in Chinese schools, and scholars in China have embraced the PL concept as an important assessing standard for evaluating PE outcomes. The concept of PL has recently been interpreted by Chinese scholars as “a comprehensive disposition encompassing motivation, confidence, physical competence, knowledge and understanding to benefit the survival and development of the whole person during the life course” [8]. This is consistent with the key components of the “Whiteheadian” definition of PL. PL has also been promoted to PE teachers through continuing professional development programs held by the Education Bureau in Hong Kong [9]. Considering the multidimensional components of PL to date, the measure of actual levels of PL is especially emphasized through developing PL assessments. For example, several studies have consistently assumed that the dimensions of PL involving behavioral, psychological and physical domains were (theoretically and practically) distinct but integrated [6], and “cannot be assessed / measured in a traditional and conventional sense using simplistic and linear methods” (p.681) [10]. Therefore, an appropriate physical skill evaluation combining all parameters of PL was required to meet this need in response to which Longmuir and colleagues have developed the Canadian Assessment of Physical Literacy (CAPL) [11]. CAPL is the first valid, reliable and comprehensive protocol in Canada for monitoring actual levels of PL among Canadian children [11]. The development progress of the assessment underwent a three-round Delphi expert review and then confirmed the model as the CAPL [12]. Recently, the research group has updated the model in a second edition, the CAPL-2. This is a more streamlined version that aims to be more easily utilized in schools by PE teachers [13]. The four domains of CAPL-2 are Physical Competence, Daily Behavior, Knowledge and Understanding, and Motivation and Confidence. Numerous studies have adopted CAPL/CAPL-2 in exploring children’s actual PL levels and the relationship between their PL and physical activity [14–16].

PL perceptions may also be important for children’s physical activity. Perceived physical literacy (perceived PL) has been constructed through the development of the Perceived Physical Literacy Instrument (PPLI) in PE teachers and adolescents within the educational setting [17, 18]. The validated instrument identified perceived PL as an individual’s perceived capability in pursuing a healthy and active lifestyle [19], which includes three attributes: (1) *Knowledge and Understanding*; (2) *Self-expression and Communication with others*; and (3) *Sense of self and Self-confidence*. Choi and colleagues have demonstrated the predictive effect of perceived PL attributes on physical activity levels among Hong Kong adolescents [20]. They assume that the attribute of *Knowledge and Understanding* is held by individuals who describe and evaluate their movement experiences and physical activity lifestyle and how to improve their movement and all-round health; the attribute of *Self-expression and Communication with others* could encourage individuals to react to interpersonal interactions through feeling and responding while participating in physical activity; while the attribute of *sense of self and self-confidence* enables adolescents to gain rewarding physical activity experiences to develop on their PL journey. This is partially supported by the research in which a significant positive association was reported between physical activity and perceived PL in adolescents and these attributes generated a variance of 5.2% in physical activity levels [20].

Given that children with low actual PL levels and low PL perceptions are less likely to participate in physical activity compared to their physically-literate peers [15, 20], it is important to understand how perceptions might be related to actual PL. However, no research has explored the associations between actual PL and the perceptions of PL, in spite of the importance of PL in predicting children’s physical activity levels. For the purposes of understanding how well children’s PL perceptions could reflect their actual PL levels, it is important that the constructs are aligned in a similar way. As such, the attributes of the perceived PL, including *Knowledge and Understanding, Self-expression and Communication with others, and Sense of self and Self-confidence,* have been reinforced with the core attributes of motivation, confidence, physical competence, and interaction with the environment emphasized in Whitehead’s (2010) definition. The perceived PL has been echoed with several of the kernel attributes of the PL concept [4]. Children develop their level of PL through embodied experiences as they grow during their life journey. CAPL provides an overall PL assessment for 8- to 12- year-old children. In Hong Kong, this age group of children are primary school aged children. Current assessment methods of PL have been intended mainly for use in the school environment [21]. In sport and education systems, children are mostly grouped by age as an administrative strategy for comparing age-adjusted competition and learning opportunities [22, 23]. However, research has indicated that age grouping by a certain cut-off date promotes a relative age difference which has an impact on children who are born on the “early side” of the cut-off date [24]. The differences are known as the relative age effect (RAE) bias [25, 26]. A great deal of research has shown that RAE is associated with children’s performance in the testing of fitness and fundamental movement skills [24, 27]. Baker, Horton, Robertson-Wilson, and Wall have also assumed that RAE works as a maturational factor to influence an athlete’s development in the elite level [28]. Since the attributes of physical competence and motor skills and fitness tests were included in CAPL-2 assessments, the influence of RAE on children’s objective PL and perceptions of PL should be considered [29]. Despite the important influence RAE generates in the development of children’s PL, few studies have examined the potential effect of RAE on the PL perceptions and the child’s actual PL levels. Understanding the relationship between actual level of PL and perceived PL, and how associations may differ according to their RAE and by sex, could inform interventions aiming to improve children’s PL, and therefore hopefully decrease health risks related to physical inactivity.

The purpose of this study was to explore the associations between perceived and actual levels of PL in primary school-aged Chinese students, and to examine RAE, gender differences and interaction effect (2X4) in perceived and actual levels of PL.

## Methods

### Participants

Participants were 327 primary students (153 boys, 174 girls; M_age_ = 10.0) recruited from two public schools in Shenzhen and Hong Kong SAR, located in the South of China. All the participants were convenience samples of children attending schools, and whose parents or legal guardians provided written consent for their children to cooperate with our research. Although adopting a convenience sampling, recruiting students from public schools in China could provide a representative status for this study, namely that the school size, schedule of class and regular physical education could represent most of the primary schools in China.

### Measures

Children’s perceived PL was assessed by the adolescents’ version of PPLI, a nine-item questionnaire consisting of three attributes: *Knowledge and Understanding* (e.g. “I have a positive attitude and interest in sports”), *Self-expression and Communication with others* (e.g. “I am capable of handling problems and difficulties”), and *Sense of self and Self-confidence* (e.g. “I possess self-management skills for fitness”). To be more specific, *Knowledge and Understanding* examined whether an individual’s acquisition of knowledge and understanding contributed towards the benefits of being physically literate; the dimension of *Self-expression and Communication with others* monitored levels of physical literacy when an individual expresses himself or communicates with the environment through physical activities; *Sense of self and Self-confidence* related to the participant’s sense of self and his/her self-confidence when participating physical activities [18]. Each response was rated on a 5-point Likert scale, ranging from strongly disagree to strongly agree. Adapted from a previous version from PE teachers, the current questionnaire was proved to be valid through Confirmatory Factor Analysis (CFA), which showed chi-square (*χ*^*2*^ = 321.54, *df* = 24, *p* < .05), CFI = .95, RMSEA = .08, SRMR = .04 [17]. The questionnaire also showed an acceptable reliability with α values ranging from .68 to .76.

Children’s actual PL was assessed using the Canadian Assessment of Physical Literacy, second edition (CAPL-2) [13]. The CAPL-2 is claimed to be the first comprehensive protocol that can accurately and reliably assess a broad spectrum of skills and abilities that contribute to and characterize the physical literacy level of a participating child [30]. The assessment tool includes four domains: Daily Behavior, Physical Competence, Knowledge and Understanding, and Motivation and Confidence. The total score for a child’s PL level is 100.

Daily Behavior contained two parts: objectively-measured step counts and self-reported moderate-to-vigorous physical activity (MVPA) (i.e., number of days a week that a child engaged in activities that made them breathe harder and their heart beat fast). Step counts were measured by the ActiGraph GT3X+ accelerometers [31] worn on the waist for children for seven consecutive days. The monitor wear time was at least 10 h/day for a minimum of 4 days according to the CAPL-2 manual, and was removed only during water activities (i.e., showering or swimming). The total score of Daily Behavior assessment was 30 points. Physical Competence contained three parts: i) FitnessGram 15 m/20 m PACER (Progressive Aerobic Cardiovascular Endurance Run) [32] for evaluating aerobic fitness; ii) Plank Assessment of Torso Strength [33] for testing musculoskeletal endurance, which is related to back health, the ability to stabilize the body, and the function of both the upper and lower limbs; and iii) the Canadian Agility and Movement Skill Assessment (CAMSA) for assessing motor competence [34]. The total score of Physical Competence assessment was 30 points. Knowledge and Understanding assessed a child’s PL-related knowledge, with five questions equaling a total of 10 points. The Motivation and Confidence domain evaluated a child’s confidence in his/her ability to be physically active and his/her motivation to participate in physical activity. A revised version of “What’s most like me” (CSAPPA) questionnaire [35] was adopted for assessing this domain with a total of 30 points. The CAPL was reported to be a valid and reliable instrument to use for children aged 8 to 12 years old, with a chi-square (*χ*^*2*^ = 98.63, *df* = 38, *p* < .05) showing acceptable fit, CFI = .96, RMSEA = .06, and the CAPL-2 was also evaluated adequately for model fit after revisiting the concept of PL [13].

### Procedures

Two professional translators worked on the cross-cultural translation of the manual of CAPL-2 [13]. A double-back reverse independent translation procedure was guaranteed [36]. This translation required an independent translation from English to traditional Chinese, and then back translation was also completed. The translators did not have access to the original English version of the manual. Further revision was made by a panel of experts in PE after checking the translated version of the manual.

Upon receiving the ethical approval granted by the University Survey and Behavioral Research Ethics Committee at the Chinese University of Hong Kong, invitation letters were sent to school principals. Informed consent forms were collected from parents or guardians. As the manual recommended, two appraisers with both genders (male and female) were needed for evaluating the aerobic test, motor skill test and muscular endurance test. Hence, an appraiser training workshop was conducted to set a consistent scoring system before the data collection. Appraisers were recommended to have valid First Aid, Cardiopulmonary Resuscitation (CPR) training and a valid Criminal Records Check.

Data collection included the following phases: On the first testing day, the participants were required to complete CAMSA, Plank, Knowledge and Understanding questionnaires and PPLI. Upon completing the instruments, participants were divided into two groups with one appraiser per group and rotated around the stations (one test per station) until the assessment was completed. Prior to CAMSA, children watched the test presentations twice performed by one appraiser. During the first presentation, the presenter moved slowly through the entire course with a detailed verbal description of each skill. For the second time, the demonstration was at full speed while the appraiser maintained skill accuracy. Participants all needed to practice twice with full speed while maintaining their skill accuracy. The higher score combining time and skill was taken into account for his/her final grade. In the Plank test, participants watched demonstrations and stopwatches were started to record when they had reached the right postures. One warning was announced when their positions were too low/high or unable to hold. The recording time was stopped when their postures moved a second time.

At the second school visit, children participated in the PACER 15 m/20 m shuttle run for monitoring aerobic fitness after completing the Motivation and Confidence questionnaire. Due to limited space, all participants ran from one marker to another marker set 15 m apart, while keeping pace with a prerecorded Cantonese cadence. The total number of laps achieved by the participants was recorded and then conversed to the standardized 20 m PACER score using FitnessGram PACER Conversion Chart [32]. Lastly, children were distributed ActiGraph GT3X+ accelerometers for measuring step counts for seven consecutive days. Actual and perceived PL measurements took place among the children during their scheduled PE lessons.

### Data analysis

Participants were not included for further data analysis unless they had fully completed each domain of CAPL-2. The missing values belonging to the Physical Competence domain could be calculated according to the fraction that the CAPL-2 manual provided. A maximum of one protocol could be completely missed and still have a calculated score [13]. In total, 237 out of 271 participants who completed the whole CAPL-2 assessments were included for further analysis. Descriptive statistics were calculated for the perceived and actual levels of PL among all the participants. Considering the relative age effect (RAE), the participants were stratified into four groups as follows: Quarter 1 (Jan-Mar), Quarter 2 (Apr-Jun), Quarter 3 (Jul-Sep), and Quarter 4 (Oct-Dec) [29]. Pearson correlations were calculated between each attribute of PL perceptions and actual PL among boys and girls. Multivariate analysis of variance (MANOVA) was computed to determine whether the between-gender and between-RAE differences existed in the PL perceptions and actual PL. The interaction effect of 2 × 4 (gender x RAE) on all the attributes was also calculated. Data analyses were conducted using SPSS (version 23).

## Results

A total of 327 participants (153 boys, 174 girls; Mage = 10.0) completed all the assessments or met the criteria for calculating the missing values. Table 1 reported the birth month distribution of the sample. Table 2 showed the value of mean, SD, min and max divided by gender, and RAE. Pearson’s correlation coefficients among all the variables separated by gender were shown in Table 3.

**Table 1:** Participants’ birth distribution in regards to the relative age effect (*N* = 327)

Category	Birth month	*n*	Percentile (%)
Quarter 1	Jan to Mar	69	21.1
Quarter 2	Apr to Jun	70	21.4
Quarter 3	Jul to Sep	88	26.9
Quarter 4	Oct to Dec	100	30.6

**Table 2:** Descriptive statistics for attributes of perceived and actual level of PL separated by gender and relative age level (*N* = 327)

Variable	Boys				Girls				Q1	Q2	Q3	Q4
	*M*	*SD*	*Min.*	*Max.*	*M*	*SD*	*Min.*	*Max.*	*M (SD)*	*M (SD)*	*M (SD)*	*M (SD)*
KU_PPL (1–15)	13.0	2.1	7.0	15.0	12.7	2.0	5.0	15.0	13.0 (2.2)	12.3 (2.0)	12.8 (2.1)	13.1 (2.0)
SE_PPL (1–15)	11.9	2.7	3.0	15.0	11.9	2.1	6.0	15.0	12.2 (2.5)	11.6 (2.2)	11.8 (2.5)	12.1 (2.4)
SS_PPL (1–15)	12.3	2.5	5.0	15.0	11.9	2.3	5.0	15.0	12.0 (2.6)	11.9 (2.0)	12.0 (2.4)	12.3 (2.4)
KU_CAPL2 (0–10)	5.6	2.4	0.0	10.0	5.8	2.3	0.0	10.0	5.5 (2.4)	5.4 (2.4)	6.1 (2.2)	5.6 (2.5)
MC_CAPL2 (0–30)	22.6	4.3	10.1	30.0	21.9	4.7	6.6	30.0	21.1 (5.3)	22.2 (3.7)	22.8 (4.4)	22.6 (4.5)
DB_CAPL2 (0–30)	13.0	4.7	4.0	26.0	10.3	3.8	3.0	24.0	11.7 (4.1)	11.2 (4.1)	12.1 (4.7)	11.3 (4.5)
PC_CAPL2 (0–30)	17.6	5.6	4.9	28.4	17.1	4.4	7.6	26.9	17.0 (4.6)	17.1 (5.0)	18.0 (5.2)	17.1 (5.2)
PPL (1–45)	37.3	6.5	20.0	45.0	36.5	5.6	18.0	45.0	37.2 (6.5)	35.8 (5.4)	36.7 (6.3)	37.5 (6.0)
CAPL2 (0–100)	58.9	11.0	34.9	90.3	55.1	9.8	28.4	78.4	55.3 (10.4)	55.9 (8.4)	59.1 (10.7)	56.7 (11.6)

*Notes*. KU_PPL, SE_PPL, and SS_PPL were three attributes of Perceived Physical Literacy; KU_CAPL, MC_CAPL, DB_CAPL, and PC_CAPL were four domains of actual Physical Literacy; PPL = total score of Perceived Physical Literacy; CAPL2 = total score of actual Physical Literacy measured by CAPL2

**Table 3:** Pearson’s correlation coefficients for attributes of perceived and actual level of PL divided by gender (*N* = 327)

Variable	1	2	3	4	5	6	7	8	9
(1) KU_PPL									
Boys	–								
Girls	–								
(2) SE_PPL									
Boys	.63^**^	–							
Girls	.61^**^	–							
(3) SS_PPL									
Boys	.70^**^	.77^**^	–						
Girls	.66^**^	.69^**^	–						
(4) KU_CAPL2									
Boys	.14	.16^*^	.10	–					
Girls	.15	.15^*^	.18^*^	–					
(5) MC_CAPL2									
Boys	.36^**^	.30^**^	.38^**^	.15	–				
Girls	.49^**^	.37^**^	.42^**^	.10	–				
(6) DB_CAPL2									
Boys	.31^**^	.20^**^	.31^**^	.01	.23^**^	–			
Girls	.16^*^	.14	.14	.04	.27^**^	–			
(7) PC_CAPL2									
Boys	.25^**^	.24^**^	.32^**^	.11	.33^**^	.22^**^	–		
Girls	.11	.08	.20^**^	.41^**^	.25^**^	.10	–		
(8) PPL									
Boys	.85^**^	.91^**^	.92^**^	.15	.39^**^	.30^**^	.30^**^	–	
Girls	.86^**^	.87^**^	.90^**^	.19^*^	.49^**^	.16^*^	.15^*^	–	
(9) CAPL2									
Boys	.43^**^	.36^**^	.46^**^	.34^**^	.69^**^	.63^**^	.76^**^	.46^**^	–
Girls	.38^**^	.30^**^	.39^**^	.50^**^	.71^**^	.56^**^	.71^**^	.41^**^	–

*Notes.* KU_PPL, SE_PPL, and SS_PPL were three attributes of Perceived Physical Literacy; KU_CAPL, MC_CAPL, DB_CAPL, and PC_CAPL were four domains of actual Physical Literacy; PPL = total score of Perceived Physical Literacy; CAPL2 = total score of actual Physical Literacy measured by CAPL2

^*^p < 0.05, ^**^p < 0.01 (two-tailed)

In general, significant correlations were found between children’s perceptions and their actual level of PL in both boys (*r* = .46, *p* < .01) and girls (*r* = .41, *p* < .01). Among boys, the lowest correlation coefficients were found in the domain of Knowledge and Understanding of actual PL. Significances were only found in the total scores of CAPL-2 (*r* = .34, *p* < .01) and the attribute of *Self-expression and Communication with others* in perceived PL (*r* = .16, *p* < .05). Among girls, moderate to high correlations (*r* value from .15 to .90) were found in all the attributes with both global perceived PL and actual PL. Different from boys, a low to moderate significance in girls was found in the domain of Knowledge and Understanding with *Sense of self and Self-confidence* (*r* = .18, *p* < .05), and with Physical Competence (*r* = .41, *p* < .01). Further, girls’ correlations between *Self-expression and Communication with others* and Daily Behavior and Physical Competence were not significant according to the results.

The multivariate analyses of variance (MANOVA) tests were computed for examining the between-gender differences and between-relative age effect (RAE) on both perceived and actual levels of PL and their interaction effect (2 × 4). Post-hoc test for multiple comparisons among groups of RAE was conducted by Bonferroni adjustment [37]. For one-way MANOVA, the results showed no significant difference in both children’s perceptions of PL and actual PL based on children’s relative age effect, *F*(21, 911) = 1.37, *p* = .12; Wilk’s Λ = .92, η^2^_partial_ = .03.

The effects of gender, RAE and their interactions on the perceived PL and children’s actual PL measured by CAPL-2 were examined using two-way MANOVAs respectively. Children’s actual PL showed a significant difference of gender using Wilk’s statistics trace, *F*(4, 316) = 7.67, *p* < .001, Wilks’ Λ = .91, η^2^_partial_ = .09. No significant interaction effect was found, *F*(12, 836) = 1.19, *p* = .29, Wilks’ Λ = .96, η^2^_partial_ = .02. The follow-up univariate ANOVAs revealed that the boys’ score was significantly higher than that of their female counterparts on Daily Behavior, *F*(1, 319) = 30.15, *p* < .001, Wilks’ Λ = 540.88, η^2^_partial_ = .09. No significant gender differences were found in Knowledge and Understanding (*p* = .40), Motivation and Confidence (*p* = .32), and Physical Competence (*p* = .36).

For the perceived PL, using Wilk’s statistics trace, no significant gender differences were found on children’s perceptions, *F*(3, 317) = 1.60, *p* = .19, Wilks’ Λ = .99, η^2^_partial_ = .02; and no significant interaction effect was found, *F*(9, 772) = .47, *p* = .90, Wilks’ Λ = .99, η^2^_partial_ = .004. Boys scored slightly higher in all the attributes than girls but no significant differences were shown. Girls only acquired a higher score on *Knowledge and Understanding* in Q2 (*M* = 12.31) compared to boys (*M* = 12.31), and *Self-expression and Communication with others* in Q1 (*M* = 12.24) compared with boys (*M* = 12.22).

## Discussion

This study was the first to explore the associations between the perceived and actual level of PL among Chinese primary school children and to examine the possible gender and relative age differences among those children. The results revealed that there were significant relationships between children’s perceived and actual level of PL in Chinese primary school children. Gender differences on actual PL, especially on the Daily Behavior domain of actual PL among Chinese children, were also observed. However, significant differences were not found on RAE in all the variables of this studied population. No interaction effect (gender x relative age effect) was found in all the associations among children.

Notwithstanding no previous studies for exploring the relationship between perceived and objectively measured PL, the results from the current study provides empirical evidence that the two perspectives have inter-related and significant associations. While similar studies in a related field have offered a number of evidence-based or theoretical explanations, they have assumed that children’s perceptions of their abilities closely approximate their actual skill competence [38] and a few studies have found significant relationships between perceived and actual fundamental movement skills [39, 40], and objective and subjective-measured physical activity [41], etc. As an integrated concept emphasizing the embodiment that encompasses “the motivation, confidence, physical competence, knowledge and understanding” as factors for engagement in physical activity, perceived and actual levels of PL theoretically, and indeed empirically, have proven to be significantly associated. Furthermore, the results indicate the importance of considering an individual’s perceptions of PL when assessing his/her actual PL level [2].

While perceived PL is feasible and practical to assess in a normal school environment, the significant associations between the perceived PL and actual levels of PL can provide a different angle for measuring students’ PL level. Given that children’s perceptions can directly reflect their actual PL level based on the assumed interpretation, Perceived Physical Literacy Instrument (PPLI) would be a more convenient tool to use in the educational environment [17, 18]. Currently, PPLI has already been adopted in Hong Kong secondary schools to examine the effect of teaching intervention on adolescents’ PL and learning outcomes [2]. Other evidence has also proved that perceived PL is positively associated with adolescents’ physical activity levels, especially within recreational physical activity [20]. According to Whitehead, the philosophical foundations of the concept of PL concerning monism and embodiment support the belief that the body and mind are one and cannot be separated [4, 21]. PL refers to the interconnected and inseparable nature of all the capabilities, that is, thoughts, feelings, and movements are intricately correlated [21]. This partly explains that the PL perceptions of an individual’s psychological domain have unified inseparably with the actual PL level, or their actual physical activity patterns [20].

The present study has also investigated gender differences, relative age bias, and their interaction effect on each dimension of perceived and actual levels of PL. Although the results only show that gender differences exist in the Daily Behavior domain of actual PL, they are consistent with previous studies that have found that boys score slightly higher than girls in most domains of CAPL-2 tests [11, 42]. Although the manual recommended pedometers for measuring children’s step counts objectively, the current studies have adopted ActiGraph GT3X+ accelerometers for step evaluation [31]. One of the main reasons for choosing this device is because it can measure various intensity of physical activity during a seven interval, and not focusing only on steps [43]. Moreover, research has indicated that the accuracy of ActiGraph GT3X+ accelerometers can be granted due to set instrument sensitivity thresholds and/or attachment compared to pedometers [44]. Therefore, the significant low level of Daily Behavior score in this study may result from the different devices used in monitoring step counts. Research related to gender differences in physical activity also provided evidence that boys are more active than girls in relation to step counts, MVPA and overall physical activity [45–47]. It is informative that there are gender differences in each domain of the objectively measured PL, and to consider encouraging girls’ participation in more activities, as they showed a relatively less active lifestyle during their earlier years [48].

Although a number of studies have found that RAE bias exists in elite-level athletes’ development [28] and motor abilities in youth population [49], relative age differences are not observed significantly in the perceived or actual levels of PL. These findings appear to be similar with a previous study exploring RAE bias on Canadian children’s objective PL. The study conducted by Dutil et al. [29] has concluded that RAE bias is negligible among all the domains and overall PL levels. In assessing objective PL, the performance of children born in the months preceding the school entry cut-off date were not influenced by the relative age difference compared with their counterparts born later in the same year [29]. However, a previous study has found that the RAE bias will influence the results of anthropometric measurements in children. However, this difference does not appear to have much influence on the actual PL levels as proven by the scholars [29]. The research that has focused on relative age differences is relatively little and previous studies have suggested that the RAE is not regarded as an important factor when monitoring actual PL level of children with CAPL-2 [27, 29].

For the perceived level of PL, no significant results of gender or RAE differences are found in the attributes of perceived PL. However, boys perceive more PL than girls in all the attributes, which is also consistent with previous studies [19, 20]. The findings are expected, as previous research has shown that girls tend to participate less in physical activity [42, 50], and with older age groups preferring non-physical activity-based pursuits that will influence their perceptions of PL imperceptibly. Researchers are challenged with exploring how to motivate children to move and to match their perceived ability in pursuing a physically active lifestyle [42]. Therefore, the study has particularly drawn attention on the important role that perceived PL plays in the current challengeable environment in which children, especially females, are becoming less active. Although complicated, the concept of PL and the perceived ability of acknowledging PL highlight that the associations between the actual and perceived levels of PL should be highly valued for maintaining a physically active lifestyle [4].

Despite valuable strengths being mentioned above for perceived and actual PL, the study is not without its limitations. First, the instrument of PPLI primarily does not include the dimension of perceived competency although PPLI is credited as a reliable measurement for assessing perceived PL. Concluding from the current study, perceived PL is still in the development stage and few studies have reported the association between PL perceptions with physical activity [20]. This study serves as a pioneer to explore the possible relationship between children’s actual PL and perceptions to acquire a broader understanding of PL. The suggestions for exploring a comprehensive and streamlined instrument for assessing perceived PL should be particularly considered for further research on perceived PL. For example, perceived motor competence has been well assessed through a pictorial format of a self-perception profile of children identical to actual movement skills objectively measured [39, 51]. However, unlike movement skills, PL is an abstract conceptualized construct that compasses a wide variety of dimensions. A more specific instrument for assessing perceived PL is not easy to develop, yet such aninstrument is urgently needed for acquiring a boarder understanding of PL.

As a battery of standardized assessment protocols to measure actual PL objectively, CAPL-2 is regarded to be valid and reliable to assess children’s actual level of PL. Notably, the comprehensive assessments include self-perceived measures in the Knowledge and Understanding domain and Motivation and Confidence domain of CAPL-2. However, the multidimensional concept of PL requires an objective assessment that encompasses and combines multiple components of PL, part of which cannot be directly measured, such as motivation, confidence, knowledge and understanding [6, 13, 30]. Edwards et al. have also argued that measuring PL needs to combine all parameters of PL with qualitative and quantitative methods [10]. The suggestions seem inspiring only from an idealist perspective, rather than in a practical school environment. A number of studies have reported that PE teachers intend to leave their profession due to high workload and scarcity of resources, especially in fast-paced cities like Hong Kong and Shenzhen [52–54]. As some of the measures have already been adopted by PE teachers in schools (such as PACER, Plank), CAPL-2 improves with its second edition to provide a streamlined and convenient assessment battery for PE teachers to use without adding an extra burden. Using CAPL-2 for monitoring the actual level of PL for the current study is a compromise and takes the above factors into consideration.

Last but not least, the present study is designed as a cross-sectional study and only collected data at a specific time point. The design may cause insufficient evidence or causal inferences for testing the associations among variables. Also, it is possible that participant’s economic background, cognitive and emotional development would influence participants’ self-reporting. However, these were not measured in this study and should be taken into account in the design of future studies [55].

## Conclusions

This study is the first to examine the relationship between perceived and actual levels of PL in Chinese children and to investigate gender, and relative age differences in the attributes of the subjective and objective PL. The results show that the perception of PL is significantly correlated with actual level of PL in school-aged children in China. Moreover, gender differences remain a factor on the Daily Behavior domain of actual PL, and relative age bias does not play an important part for both actual PL and the perception of children. This study highlights the importance of perceived PL and its significant associations with children’s actual PL, which implies that the perceived and actual levels of PL are interconnected to allow an easier approach when monitoring an individual’s PL level. Additional attention should be given to girls considering they had relatively low score of PL acquired in their early childhood. Relative age effect is negligible with regard to the attributes of the perceptions and actual PL level. Further studies should focus on adopting a longitudinal approach to monitor children’s perceived and actual PL levels.

## Data Availability

The data generated during this study are not public because availability was not included in the study plan approved by the ethics committee and in the informed consent obtained from the participants. However, the data are available from the corresponding author on reasonable request.

## Abbreviations

CAMSA: Canadian Agility and Movement Skill Assessment
CAPL: Canadian Assessment of Physical Literacy
CAPL-2: Canadian Assessment of Physical Literacy, Second Edition
FMS: Fundamental movement skills
MANOVA: Multivariate analysis of variance
MVPA: Moderate-to-vigorous physical activity
PA: Physical activity
PACER: Progressive Aerobic Cardiovascular Endurance Run
PE: Physical education
PL: Physical literacy
RAE: Relative age effect
